# Mitochondria-Associated Pathways in Cancer and Precancerous Conditions: Mechanistic Insights

**DOI:** 10.3390/ijms26178537

**Published:** 2025-09-02

**Authors:** Ling Li, Dan Pan, Ruixue Ai, Yu Zhou

**Affiliations:** 1 State Key Laboratory of Oral Diseases, National Center for Stomatology, National Clinical Research Center for Oral Diseases, Research Unit of Oral Carcinogenesis and Management, Chinese Academy of Medical Sciences, West China Hospital of Stomatology, Sichuan University, Chengdu 610041, China; 2 Department of Clinic Molecular Biology, University of Oslo and Akershus University Hospital, 1478 Lørenskog, Norway

**Keywords:** mitochondria disease, cancer, precancerous condition, gene mutation, mitochondrial dysfunction

## Abstract

Mitochondria perform critical roles in cellular functions, particularly in metabolism and cell death regulation. Mutations in nuclear and mitochondrial genes can cause mitochondrial dysfunction, leading to classical mitochondrial diseases. Emerging evidence suggests that mitochondrial adaptations in cancer support the high energy demands of proliferating cells and contribute to tumor progression through anti-apoptotic mechanisms, dysregulated mitochondrial quality control (mtQC), and altered mitochondrial DNA (mtDNA) copy numbers. Interestingly, several mitochondrial pathways involved in cancer progression resemble those implicated in mitochondrial diseases. From this perspective, although cancer is not a classical mitochondrial disease, its progression involves mitochondria-associated pathways similar to those in mitochondrial disorders, suggesting that cancer may be considered a mitochondria-related disease in a broader sense. Understanding these shared mechanisms could provide new insights into precision treatment strategies. Furthermore, mitochondrial dysfunction is increasingly recognized in precancerous conditions, suggesting its potential as a target for early intervention. Oral potentially malignant disorders (OPMDs) serve as a valuable model for studying these mitochondria-associated mechanisms, offering a promising avenue for both therapeutic advancements and preventive approaches.

## 1. Introduction

Mitochondrial diseases refer to a class of genetic disorders characterized by dysfunctional mitochondria [1]. Cancer, on the other hand, is typically associated with the accumulation of genetic mutations that lead to cellular transformation [2]. Given the essential role of mitochondrial function in cellular homeostasis, investigating how mitochondrial dysfunction contributes to cancer biology is highly relevant. Research into mitochondrial dysfunctions within the context of cancer has been ongoing for several decades (Figure S1). Most studies focus on mitochondrial dysfunction in cancer metabolism. Here, we further summarize its roles in cell death regulation, mitochondrial quality control (mtQC), and mitochondrial DNA (mtDNA) copy number changes to provide a more comprehensive understanding of mitochondria-associated mechanisms in cancer, which may inform mitochondria-targeted clinical strategies.

Furthermore, improving prognosis and advancing cancer prevention highlight the need to investigate mitochondria-associated mechanisms in precancerous conditions. A precancerous condition is “a generalized state associated with a significantly increased risk of cancer” [3]. Based on this concept, we broadly discuss mitochondrial dysfunction that occurs prior to malignant transformation. Effective treatment during this phase can potentially reverse the condition and prevent cancer development. Therefore, investigating mitochondria-associated alterations during the precancerous stage is crucial. However, current research in this area remains limited. In this review, we summarize existing evidence on mitochondrial involvement in precancerous conditions to inform future studies.

## 2. Mitochondrial Dysfunctions in Cancer Progression

### 2.1. Oncogenic Mutations in nDNA Lead to Reprogramming of Mitochondrial Metabolism

In contrast to the metabolic processes of differentiated cells, proliferating cells undergo strategic adaptations to prioritize the efficient uptake and incorporation of essential nutrients into biomass to sustain vigorous proliferation [4]. In cancer cells, mitochondria play a core role in orchestrating metabolic reprogramming. This reprogramming enables cancer cells to acquire and utilize nutrients in a manner conducive to proliferation through the biosynthesis of macromolecules, rather than focusing solely on efficient ATP production. Here, we elucidate the genetic mechanisms underlying mitochondrial-associated metabolism and their roles in cancer progression.

#### 2.1.1. Glucose Metabolism Alterations: From Complete Oxidation to Aerobic Glycolysis

**Figure 1:**
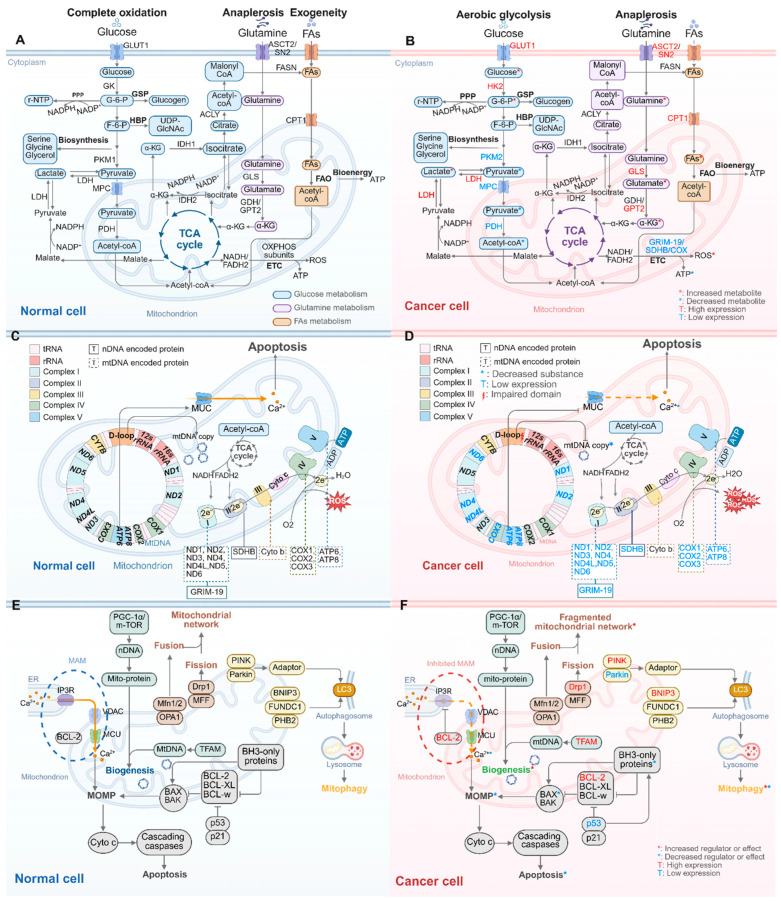
Mitochondrial alterations induced by oncogenic mutations in nDNA and mtDNA: A comparison between normal and cancer cells. (**A**,**B**) Mitochondrial metabolic alterations due to nDNA mutations. In normal cells, glucose, glutamine, and FAs undergo complete oxidation through the TCA cycle and the ETC, generating ATP and maintaining proper ROS levels. In cancer cells, aerobic glycolysis dominates glucose metabolism, diverting glucose carbon away from the TCA cycle to fuel biosynthetic pathways. Increased glutamine anaplerosis replenishes TCA cycle intermediates, while elevated FA transport enhances acetyl-CoA supply for the TCA cycle. Impairments in ETC subunits, such as SDHB and GRIM-19, reduce ATP production and elevate ROS levels. (**C**,**D**) Alterations in OXPHOS, mtDNA copy number, and apoptosis due to mtDNA mutations. In normal cells, mtDNA-encoded genes regulate key mitochondrial functions, including metabolism, mtDNA replication, and calcium homeostasis. In cancer cells, mutations in OXPHOS subunits—such as ND1, ND2, ND4, ND4L, ND6, COX3, and ATP6/8—lead to defective OXPHOS and excessive ROS production. Mutations in the D-loop region reduce mtDNA copy number, while ATP6 mutations suppress apoptosis. (**E**,**F**) Alterations in mitochondria-associated apoptosis and mtQC due to nDNA mutations. In normal cells, calcium released from the ER into the mitochondrial matrix and BCL2 family proteins regulate apoptosis. mtQC is maintained through coordinated regulation by nuclear and mitochondrial proteins. In cancer cells, BCL-2 overexpression inhibits IP3R, reducing ER-to-mitochondria Ca^2+^ flux, while TP53 mutations and p53 downregulation enhance BAX suppression, collectively preventing apoptosis. Additionally, mutations in *PARK2* and reduced *Parkin* expression impair mitophagy. Other regulators, including *TFAM*, *Drp1*, *PINK1*, and *BNIP3*, are upregulated through oncogenic transcriptional or post-translational modifications, reprogramming the mtQC process in cancer cells. GLUT1, glucose transporter 1; GK, glucokinase; HK2, hexokinases II; VDAC, voltage-dependent anion channel; G-6-P, glucose-1-phosphate; F-6-P, fructose-6-phosphate; GSP, glycogen synthesis pathway; PPP, pentose phosphate pathway; rNTP, ribonucleotide triphosphate; HBP, hexosamine biosynthesis pathway; UDP-GlcNAc, Uridine diphosphate N-acetylglucosamine; PKM1/2, pyruvate kinase M1/2; LDH, lactate dehydrogenase; PDH, pyruvate dehydrogenase; PDK, pyruvate dehydrogenase kinase; NADH, nicotinamide adenine dinucleotide; FADH2, flavin adenine dinucleotide; ETC, electron transport chain; α-KG, α-ketoglutarate; GDH, glutamate dehydrogenase; GPT2, glutamate pyruvate transaminase 2; GLS, glutaminase; IDH, isocitrate dehydrogenase; ACLY, ATP citrate lyase; FASN, fatty acid synthase; CPT1, carnitine palmitoyltransferase 1; FAs, fatty acids; FAO, fatty acids oxidation. ND, NADH dehydrogenase; CYB, cytochrome b; COX, cytochrome c oxidase; ATP6/8, ATP synthase F0 subunit 6/8; MUC, mitochondrial unspecific channel. ER, endoplasmic reticulum; IP3R, inositol 1,4,5-triphosphate receptor; MCU, mitochondrial calcium uniporter; MAM, mitochondria-associated ER membrane; MOMP, mitochondrial outer membrane permeabilization; m-TOR, mammalian/mechanistic target of rapamycin; TFAM, transcription factor A, mitochondrial; Mfn1/2, mitofusin 1/2; OPA1, optic Atrophy 1; MFF, mitochondrial fission factor; FUNDC1, FUN14 domain containing 1; PHB2, prohibitin 2; NIX, also BNIP3L, BCL2 interacting protein 3 like; LC3, also MAP1LC3A, microtubule associated protein 1 light chain 3 alpha.

In quiescent cell, glucose initially undergoes fermentation to form pyruvate within the cytoplasm. In the presence of oxygen, pyruvate is transported into the mitochondria, where it acts as the primary fuel for the TCA cycle. Within this cycle, acetyl-CoA derived from pyruvate is oxidized, yielding ATP and by-products. These by-products, such as nicotinamide adenine dinucleotide (NADH) and flavin adenine dinucleotide (FADH2), subsequently contribute electrons to the electron transport chain (ETC), fostering substantial ATP generation. In the absence of oxygen, pyruvate is converted into lactate by lactate dehydrogenase (LDH), with minimal ATP production, a process known as anaerobic glycolysis (Figure 1A). In cancer cells, glucose undergoes aerobic glycolysis, a process in which it is converted to lactate, producing relatively low ATP yields despite the presence of oxygen [5]. This Warburg effect in cancer cell encompasses two main components: increased glycolysis and decreased oxidation, resulting in rapid glucose utilization and increased biomass through glycolytic branching pathways.

On one hand, glycolysis in the cytoplasm is upregulated by abnormal glycolytic enzymes resulting from nDNA oncogene mutations (Figure 1B, Table S1). The overexpression of oncogenic *c-myc* (encoding c-Myc, an oncogenic transcription factor) enhances the transcriptional rate of glucose transporter 1 (GLUT1), accelerating glucose uptake [6]. Hexokinase II (HK2) is expressed at significantly higher levels compared to hexokinase IV (glucokinase, GK) and directly binds to the voltage-dependent anion channel (VDAC) on the outer mitochondrial membrane (OMM). This interaction facilitates the rapid phosphorylation of glucose to glucose-6-phosphate (G-6-P) using ATP passing through the pore [7]. The phosphorylated glucose is retained within the cell due to the added charge [8], maintaining a high intracellular glucose concentration in cancer cell. High expression levels of HK2 can result from the co-mutation of tumour suppressor genes *Pten* and *p53* in mice [9]. Mechanistically, Pten deletion enhances HK2 mRNA translation via the AKT-mTORC1-4EBP1 axis, while p53 loss stabilizes HK2 mRNA by inhibiting miR143 biogenesis [9]. Pyruvate kinase M2 (PKM2) is a less active isoform of PKM, which catalyses the final step in glycolysis and produce pyruvate. c-Myc upregulates the transcription of heterogeneous nuclear ribonucleoprotein (hnRNP) proteins hnRNPI, hnRNPA1, and hnRNPA2, resulting in a high PKM2/PKM1 ratio and promoting glycolysis accumulation in human gliomas [10]. Reduced pyruvate production shifts glucose metabolites from energy production to anabolic processes in cancer cells. Notably, the upregulation of LDH leads to a decrease in pyruvate and an increase in lactate conversion [11], which helps to maintain the glycolysis process.

On the other hand, nuclear-encoded mitochondrial proteins regulated by oncogenes can inhibit the processes of pyruvate transport into mitochondria and its subsequent oxidation (Figure 1B, Table S1). In prostate cancer cells, chicken ovalbumin upstream promoter-transcription factor II (COUP-TFII) inhibits the promoter activity of mitochondrial pyruvate carrier 1 (MPC1) [12], leading to decreased mitochondrial pyruvate oxidation and a glycolytic metabolic phenotype. Regarding pyruvate oxidation within mitochondria, pyruvate dehydrogenase (PDH) is a key enzyme converting pyruvate to acetyl-CoA. Phosphorylation of PDH by pyruvate dehydrogenase kinase (PDK) leads to its inactivation. PDK2 is negatively regulated by the tumour suppressor gene *TP53* [13]. Thus, in cancer cell with *TP53* mutation, increased PDK2 activity suppresses PDH, thereby inhibiting the pyruvate oxidation process.

#### 2.1.2. Changes in Amino Acid and Lipid Metabolism: Enhanced Glutamine Anaplerosis and FAs Oxidation

In normal cells, glutamine is converted to glutamate by mitochondrial glutaminase (GLS). Glutamate is subsequently converted to α-ketoglutarate (α-KG) through reactions involving transaminases such as glutamate dehydrogenase (GDH) and glutamate pyruvate transaminase 2 (GPT2) (Figure 1A). This conversion provides nitrogen for purine synthesis and carbon units for pyrimidine, amino acid, and lipid synthesis, and this process of replenishing TCA cycle intermediates is known as anaplerosis [14]. In cancer cells, increased glutamine anaplerosis becomes a primary source of TCA cycle intermediates due to decreased availability of pyruvate-derived acetyl-CoA (Figure 1B, Table S1). Glutamine enters TCA cycle via α-KG, providing essential components for biosynthetic precursor synthesis. GLS [15] and glutamine transporters such as ASCT2 and SN2 [16] can be transcriptionally upregulated by c-Myc. Additionally, GPT2 is upregulated by *PIK3CA* mutation and increased phosphatidylinositol 3-kinase α (PI3Kα, encoded by *PIK3CA*) through PDK1-RSK2-ATF4 signalling axis in colorectal cancer cells [17].

Regarding lipid metabolism (Figure 1A), mitochondrial fatty acid oxidation (FAO) involves a cyclical series of reactions that progressively shorten FAs, generating NADH, FADH2, and acetyl-CoA with each cycle. A crucial step in FAO is the import of FAs into mitochondria, mediated by carnitine palmitoyl transferase 1 (CPT1). In cancers with *TP53* mutations, such as triple-negative breast cancer, FAO genes including *CPT1* are often overexpressed due to high c-Myc level, and then an enhanced FAs oxidation in mitochondria (Figure 1B, Table S1). However, the interaction between p53 loss and increased c-Myc requires further investigation [18].

Thus, when glucose metabolism shifts away from complete oxidation, cancer maintains bioenergetic and biosynthetic balance through increased glutamine and fatty acid supply.

#### 2.1.3. Impaired Mitochondrial OXPHOS

In the final stage of mitochondrial energy metabolism, the TCA cycle transfers electrons to the ETC in the form of NADH and FADH2 (Figure 1C). In the ETC, electrons are transferred to oxygen to generate ROS while ADP is phosphorylated into ATP. This process occurs on the inner mitochondrial membrane (IMM) and is known as OXPHOS. The ETC comprises five complexes and two electron transport carriers (coenzyme Q10 and cytochrome c), encoded by both nuclear and mitochondrial genes. Complex I (CI) and Complex II (CII) are critical for electron flow, passing electrons from TCA-generated NADH and FADH2. Electrons are transferred through NADH dehydrogenase (ND) in CI and succinate dehydrogenase (SDH) in CII, then to ubiquinone, which delivers them to Complex III (CIII) and ultimately to oxygen via Complex IV (CIV). The reduction of oxygen produces ROS. Finally, Complex V (CV), located on the IMM and containing ATP synthase, synthesizes ATP driven by the transmembrane electrochemical gradient generated by this electron transfer process.

In cancer cells, gene mutations in nDNA contribute to abnormal electron transfer in the ETC and result in excessive toxic ROS production (Figure 1B, Table S1). Toxic ROS can induce genome instability by creating an oxidative and inflammatory microenvironment and support malignant transformation [19]. Mutations in *GRIM-19* (encoding GRIM-19, a subunit of CII) were described in thyroid tumor [20]. Mutations and deficiencies in *SDHB* (encoding one of the subunits of SDH in CII) have been linked to early-onset renal cell carcinoma, with abnormal protein disrupting electron flow and increasing ROS production [21]. Additionally, p53, which transcriptionally activates the expression of SCO2 (synthesis of cytochrome c oxidase 2), is crucial for cytochrome c oxidase (COX, part of CIV). COX is a major site of oxygen utilization, hence, in p53-deficient cancer cell, COX deficiency leads to enhanced glycolysis due to ETC impairment [22].

### 2.2. Oncogenic Mutations in nDNA Disrupt the Mitochondrial Regulation of Cell Death and mtQC

Genetic events leading to metabolic reprogramming in mitochondria play a critical role in cancer progression. In addition to metabolism, mitochondrial dysfunctions in cancer cells involves mechanisms related to cell death and mtQC (including mitochondrial biogenesis, network dynamics, and mitophagy).

#### 2.2.1. Inhibition of Mitochondria-Related Apoptosis Leading to Cancer Cell Immortality

Membrane contacting sites between the endoplasmic reticulum (ER) and mitochondria form a physical platform facilitating communication between the two organelles, known as the mitochondria-associated ER membrane (MAM). MAM allows the transport of high concentrations of calcium ion (Ca^2+^) from the ER to the mitochondrial matrix. Ca^2+^ is released from the ER through the tetrameric inositol 1,4,5-triphosphate receptor (IP3R) channel, translocating across the OMM and IMM via the VDAC and mitochondrial calcium uniporters (MCUs), respectively [23,24,25]. Under physiological conditions, only a few of these channels are open, ensuring relatively low Ca^2+^ concentrations. High levels of Ca^2+^, potentially caused by the opening of non-selective large-conductance “pores”, can lead to mitochondrial dysfunction by increasing IMM permeability [25]. Excessive mitochondrial Ca^2+^ triggers the opening of the mitochondrial permeability transition pore (mPTP), promoting apoptosis by altering mitochondrial membrane permeability [26]. The regulation of Ca^2+^ flux between mitochondria and ER is mediated by the BCL-2 (B-cell lymphoma-2) protein family, located on the OMM [27]. This family includes anti-apoptotic proteins such as BCL-2, BCL-XL, and BCL-w, as well as pro-apoptotic proteins like BAX (BCL2-associated X), BAK (BCL2-antagonist/killer), and BH3-only proteins, including Bim (Bcl-2-interacting mediator of cell death), PUMA (p53-upregulated modulator of apoptosis), and Bad (BCL-2/BCL-XL-associated death promoter). The balance and interactions of these proteins, particularly the BH3-only members, play a pivotal role in initiating apoptosis [28]. Among them, PUMA is notable for its strong pro-apoptotic function and its ability to convey both p53-dependent and independent signals [29]. PUMA regulates OMM permeabilization by binding to anti-apoptotic BCL-2 family proteins with high affinity, thereby releasing pro-apoptotic effectors such as BAX and BAK from inhibition. In addition, PUMA promotes the conformational activation and oligomerization of BAX and BAK at the mitochondrial membrane [30]. BAX/BAK oligomerization can lead to OMM permeabilization, subsequently promoting apoptosis. On the other hand, BCL-2 and BCL-XL counteract pro-apoptotic Ca^2+^ signalling and apoptosis by directly targeting and inhibiting IP3R. These pro-apoptotic events culminate in mitochondrial outer membrane permeabilization (MOMP), which decreases the mitochondrial membrane potential (MMP), leading to a loss of ATP production and the release of cytochrome c (Cyto c), ultimately inducing caspase-dependent apoptosis (Figure 1E).

In cancer cells, these apoptotic pathways are suppressed (Figure 1F, Table S1). Small-cell lung cancer cell overexpresses BCL-2, which protects them from apoptosis by downregulating Cyto c release and IP3R-mediated Ca^2+^ signalling [31]. Mutations in the *TP53* gene, leading to p53 deficiency, are the most common mutations in oral squamous cell carcinoma (OSCC) [32] and occur in over 50% of human cancers [33].The p53 deficiency also serves as an anti-apoptotic mechanism, as p53 normally binds to and suppresses BCL-w and BCL-XL, in coordination with p21, to release BAX and activate pro-apoptotic functions [34].

#### 2.2.2. Increased Mitochondrial Biogenesis Providing Enhanced Mitochondrial Mass

Mitochondrial biogenesis refers to the increase in mitochondrial mass, which is a tightly coordinated process involving both nDNA and mtDNA. This process encompasses the replication and expression of mtDNA, the transport of phospholipids, and the import of nuclear-encoded proteins into mitochondria [35]. Nuclear-encoded mitochondrial proteins are activated by transcription factors such as estrogen-related receptor α (ERRα) and nuclear factor erythroid 2-like 2 (NFE2L2). The coactivator PGC-1α (peroxisome proliferator-activated receptor-gamma co-activator-1 alpha) integrates the activities of these transcription factors to predominantly regulate mitochondrial biogenesis. Among these pathways, the mammalian/mechanistic target of rapamycin (mTOR) signalling pathway can also regulate nuclear-encoded mitochondrial proteins at the translational level, serving as an upstream activator of PGC-1α [36] (Figure 1E).

In cancer cells, the key transcriptional factor involved in mitochondrial biogenesis is the oncogenic c-Myc, which targets genes such as transcription factor A, mitochondrial (TFAM), an essential activator of mitochondrial transcription and mtDNA replication [37]. Additionally, c-Myc promotes the synthesis of macromolecules like lipids and nucleotides, which support the biogenesis of cellular organelles, including mitochondria [38] (Figure 1F, Table S1). Enhanced mitochondrial mass has been linked to the propagation of stem-like cancer cells [39].

#### 2.2.3. Upregulated Mitochondrial Fission Leading to a Fragmented Mitochondrial Network

Under normal conditions, mitochondrial dynamics are regulated by a balance between fusion and fission, maintaining a homeostatic state of mitochondrial mass. In mammals, mitochondrial fusion is regulated by mitofusin 1/2 (Mfn1/2) and the mitochondrial dynamin-like GTPase optic atrophy 1 (OPA1). These dynamin-related GTPases facilitate the fusion of the outer and inner mitochondrial membranes, respectively. In contrast, mitochondrial fission is regulated by the GTPase Drp1 and its adaptors, mitochondrial fission factor (MFF) (Figure 1E). Mitochondrial fission serves to monitor poorly functioning components by either promoting mitochondrial fusion for restoration or inducing segregation for mitophagy [40].

There is no direct evidence linking fusion regulators to cancer cell. The upregulation of fission proteins has been shown to reprogram mitochondrial dynamics and create a fragmented mitochondrial network in cancer cells. In *KRAS*-induced cancer cells, mitochondrial fission is upregulated through the promotion of Drp1 serine 616 phosphorylation and Drp1 activation via the overexpression of Kras and the activation of the MAPK pathway [41] (Figure 1F, Table S1). Fragmented mitochondrial network is related to abnormal MMP and increased ROS production, driving tumorigenic transformation [41]. Mitochondrial fission is also closely linked to glycolysis, with OXPHOS inhibited in the fragmented network. Furthermore, hyper-fragmented mitochondria fail to support BAX-dependent membrane association and permeabilization, exerting an anti-apoptotic effect [42].

#### 2.2.4. Bidirectional Regulation of Mitophagy

Mitophagy is a critical component of mtQC, involving the identification and removal of severely damaged or defective mitochondria. Two main pathways exist for labelling mitochondria and delivering them to autophagosomes via interaction with LC3 (also known as MAP1LC3A, microtubule-associated protein 1 light chain 3 alpha) (Figure 1E). The first is PINK1/Parkin-dependent mitophagy, which is activated by mitochondrial membrane depolarization. This process involves the recruitment of PINK1 and Parkin to the OMM, phosphorylation of poly-ubiquitin (Ub) chains, recognition by adaptor proteins, and eventual interaction with LC3. The second pathway is Parkin-independent mitophagy, which is initiated by OMM receptors such as BNIP3, FUNDC1, and the IMM receptor PHB2. These receptors directly interact with LC3 in response to stresses such as hypoxia and oxidative stress.

Mitophagy plays a dual role in cancer. In *TP53*-mutant lung cancer, the cytoplasmic localization of p53 prevents its binding to the promoter regions of *PINK1*, resulting in elevated *PINK1* expression and enhanced mitophagy [43]. Additionally, activating *KRAS* mutations drive BNIP3-mediated mitophagy via the MAPK pathway, contributing to reduced mitochondrial content in pancreatic ductal adenocarcinoma [44]. On the other hand, in lung cancer with mutations in *PARK2* (encoding Parkin), Parkin fails to translocate onto mitochondria and is unable to recruit downstream mitophagic regulators [45] (Figure 1F, Table S1). Both excessive and insufficient mitophagy can promote cancer progression, depending on the disease stage. In early-stage cancers, impaired mitophagy leads to the accumulation of dysfunctional mitochondria, tumour-promoting ROS, and other oncogenic mitochondrial signals, such as the activation of the Warburg effect. Conversely, in established tumours, mitophagy becomes essential for tumour cell survival by clearing mitochondrial stressors, including cytosolic mtDNA and toxic ROS, thereby facilitating tumour adaptation and progression [46].

### 2.3. Mitochondrial Dysfunctions Driven by Oncogenic mtDNA Mutations

The components of the ETC are encoded by both nDNA and mtDNA. Specifically, mtDNA encodes 13 proteins that constitute CI, CIII, CIV, and CV, including NADH dehydrogenase subunits (ND1, ND2, ND3, ND4, ND4L, ND5, ND6), cytochrome b (CYB), cytochrome c oxidase subunits (COX1, COX2, COX3), and ATP synthase F0 subunits (ATP6, ATP8). Additionally, mtDNA includes two ribosomal RNAs (rRNA) and 22 transfer RNAs (tRNA), which are essential for synthesizing these complex subunits. The D-loop region in mtDNA regulates both replication and transcription of mtDNA (Figure 1C).

In cancer cells, mtDNA is especially prone to mutations due to its high exposure to ROS and its limited DNA repair mechanisms [47] (Figure 1D, Table S1). Mutations in genes encoding ND1, ND2, ND4L, ND6, COX3, ATP6 and ATP8 have been detected in childhood acute lymphoblastic leukemia. These mutations are implicated in leukemic clone development by disrupting OXPHOS function [48]. Mutations in the D-loop region have been shown to decrease mtDNA copy numbers in various cancers, including osteosarcoma [49], Ewing’s sarcoma [50], and breast cancer [51]. Additionally, mtDNA mutations is associated with the prevention of apoptosis. Mutation in *ATP6* was found to impair the activation of the yeast permeability transition pore (yPTP, also known as the yeast mitochondrial unspecific channel, yMUC) upon mitochondrial Ca^2+^ induction [52], this impairment enables cancer cell to evade apoptosis (to be elaborated later).

In light of the above, the genetic reprogramming of mitochondrial functions in cancer cells promotes tumorigenesis and progression through various mechanisms, including enhanced cancer cell proliferation, increased genomic instability, augmented tumor invasiveness, and the acquisition of cellular immortality (Figure S2). These findings underscore the critical role of mitochondrial function in cancer progression, highlighting that cancer can be comprehensively considered a mitochondria-related disease beyond its metabolic aspects. Furthermore, this provides additional potential targets for precision molecular therapies in cancer treatment (Figure S3).

## 3. Mitochondrial Mechanisms in Precancerous Conditions

Building on the identification of mitochondrial biomarkers and therapeutic targets in cancer, most of which remain at early research stages (Figure S3), we propose a detailed investigation into mitochondrial mechanisms during precancerous stages. Such studies could significantly enhance early diagnosis and cancer prevention. Here, we reviewed studies on mitochondria in precancerous conditions and summarized their pathogenetic roles, highlighting mechanisms similar to those observed in cancer. If further validated, this could extend mitochondrial-based cancer management to precancerous stages, advancing early detection and intervention.

**Figure 2:**
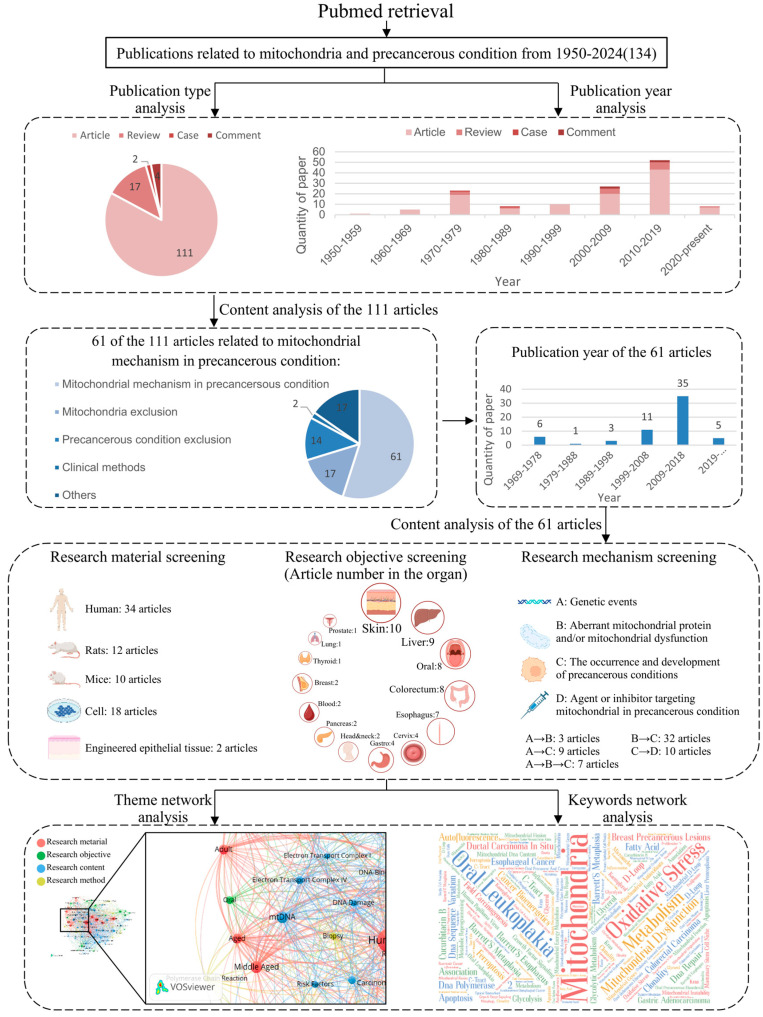
Literature review on mitochondria and precancerous conditions. A PubMed literature search using the MeSH terms “mitochondria” and “precancerous condition” identified 124 publications, with an additional 10 meeting the criteria after screening, totaling 134 for analysis. Research in this field has grown over the past few decades, predominantly comprising original studies, while recent review articles remain scarce. Among the 111 original research articles, 61 focus on mitochondrial mechanisms in precancerous lesions. Many studies involve human tissues, particularly targeting precancerous lesions of the skin, liver, oral cavity, and colorectum. Nineteen articles report genetic events linked to mitochondrial alterations. Thematic network analysis indicates that research on mitochondria in oral precancerous lesions primarily emphasizes ETC function and mtDNA. Keywords network analysis highlights “oral leukoplakia” and “oxidative stress” as frequent term.

**Figure 3:**
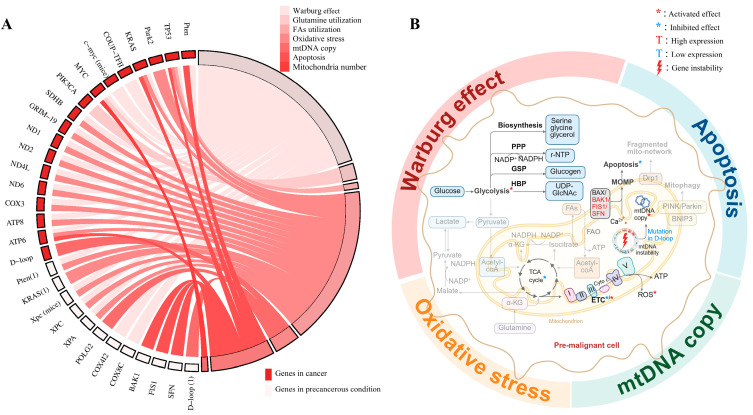
Comparison of genetic alterations and mitochondrial pathways between precancerous conditions and cancer. (**A**) Chord diagram showing the relationships between mitochondrial-related genes (inner ring) and their associated functional alterations (outer ring). The connecting bands indicate the key mitochondrial mechanisms identified in precancerous lesions—including the Warburg effect, oxidative stress, mtDNA copy number alterations, and apoptosis regulation—which mirror those observed in cancer. (**B**) Schematic illustration of the four major mitochondrial mechanisms identified in precancerous lesions. Compared with cancer, a significant portion of mitochondrial mechanisms in precancerous diseases remains unexplored (gray area).

Mitochondrial dysfunction has been implicated in the pathogenesis of various precancerous conditions, although its role remains less comprehensively understood compared to that in established cancers. Based on a literature review of the PubMed database (Figure 2), 61 articles specifically focus on mitochondrial mechanisms in precancerous lesions are summarized in Table 1. These studies show that mitochondrial dysfunction in precancerous conditions predominantly occurs through four mechanisms: the Warburg effect, changes in mtDNA copy number, apoptosis regulation, and mitochondria number (Figure 3).

Based on existing research on mitochondria-related precancerous conditions and their characteristics (Table 1), OPMD presents several properties that make it an ideal model for exploring mitochondrial pathways in pre-malignant cell: (i) High malignant transformation rate. OPMD is defined as “any oral mucosal abnormality that is associated with a statistically increased risk of developing oral cancer” [116]. Oral leukoplakia (OLK), one of the most common and extensively studied OPMD in clinical practice [116], exhibits a reported pooled proportion of malignant transformation of 7.20% (95% confidence interval: 5.40–9.10%) according to a meta-analysis conducted in 2023 [117]. This rate increases to 15.3% when epithelial dysplasia is present [118]; (ii) Clear progression process. OPMD undergoes a dynamic progression process that can be detected through tissue biomarkers and classified pathologically. For instance, OLK is diagnosed using a five-stage histopathological classification system, which includes hyperplasia, mild dysplasia, moderate dysplasia, severe dysplasia, and carcinoma in situ based on the histopathological features of epithelial dysplasia [65]; (iii) Extensive mitochondrial research and confirmed mitochondrial function. According to our literature review (Figure 2), the oral cavity has been extensively studied in mitochondrial research, with mtDNA and ETC functions emerging as promising research directions; (iv) Feasibility. The relatively accessible location of the oral mucosa compared to other lesion sites provides a distinct advantage for biopsy, thereby enhancing clinical sampling capabilities. Therefore, OPMD is chosen as the focus for further investigation into mitochondrial mechanism.

## 4. Conclusions

**Figure 4:**
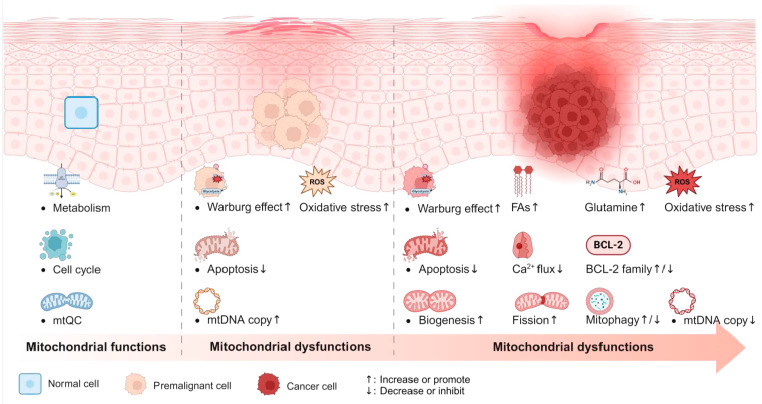
From precancerous condition to cancer: the progressive accumulation of mitochondrial dysfunction. In normal cells, mitochondria primarily regulate metabolism, the cell cycle, and mitochondrial quality control. In precancerous cells, mitochondrial dysfunction is characterized by four key alterations: enhanced Warburg effect, increased oxidative stress, inhibition of apoptosis, and an elevated mtDNA copy number. In cancer cells, these dysfunctions are further exacerbated and studied in greater detail.

In summary, we have outlined a framework summarizing mitochondrial abnormalities observed in cancer tissues and their potential mechanistic roles in tumor biology. Key changes include a metabolic shift from complete oxidation to aerobic glycolysis, enhanced glutamine anaplerosis and fatty acid utilization, decreased mtDNA copy number, and an adaptive mitochondrial network (Figure 4). These mitochondrial abnormalities underlie several hallmarks of cancer, such as enhanced proliferation, genomic instability, metastatic potential, and resistance to cell death. Importantly, several of these mitochondrial dysfunctions mirror mechanisms seen in classical mitochondrial diseases, suggesting that cancer may share mitochondria-associated pathological features. Recognizing these similarities may help uncover novel diagnostic and therapeutic opportunities. For instance, diagnostic approaches used in mitochondrial diseases, such as blood biomarkers and histochemical analyses [1], could be repurposed to identify mitochondria-related abnormalities in cancer, potentially aiding early detection. In addition, small-molecule agents targeting mitochondrial function represent a promising direction for future cancer therapies.

For early management and prevention of cancer in clinical practice, identifying mitochondrial biomarkers before malignant transformation remains crucial. Our analysis of existing research on mitochondria and precancerous conditions suggests an analogous mitochondrial-related pathological pattern in precancerous diseases (Figure 4). Discovering mitochondrial biomarkers could enhance the diagnosis and treatment of precancerous conditions, potentially preventing cancer initiation or providing palliative care to delay progression. Although mitochondrial mechanisms in precancerous conditions are not yet fully described, OPMD presents an excellent model for investigating mitochondrial pathways due to its high malignant transformation rate, its well-defined progression from OPMD to OSCC, evidence indicates that mitochondrial dysfunction is involved in OPMD progression, and the ease of tissue acquisition. Building on existing exploration on mitochondrial pathogenesis in various cancers and precancerous conditions, we can use it as a reference to further investigate our selected oral models.

By integrating insights from cancer and precancerous diseases, our study underscores the importance of mitochondria as a therapeutic target and provides a roadmap for future research aimed at leveraging mitochondrial-targeted strategies for cancer prevention and treatment.

## Figures and Tables

**Table 1:** Mitochondrial alterations in precancerous conditions.

/	Genetic Alteration	Pathway	mt-Related Protein	Alteration of mt-Function	Precancerous Conditions	Feature of Precancerous Conditions	Therapeutic Molecule and Mitochondrial Target in This Disease	Ref
nDNA	*Pten* mutation	PI3K ↑ and AMPK ↓	TCA cycle and OXPHOS gene expression ↓	Warburg effect ↑	Thyroid hyperplasia	/	/	[53]
		/	/	Oxidative stress ↑	Pancreatic precancerous lesion [54]: PanIN	Low-grade dysplasia, high-grade dysplasia (carcinoma in situ) [55]	/	[56]
	*KRAS* mutation	EGFR signalling ↑	/					[57]
	*Xpc* (mice) mutation	1. NOX1 ↑ 2. Reduced GSH/GSSG ratio ↓	1. Complex I ↓ 2. COX1, CYTB, and 16S rRNA ↑	1. Warburg effete↑ 2. Oxidative stress ↑	XP	/	TFN: DHODH ↓ [58]; Comp-1: HK2 detachment from the mitochondria [59]	[60,61,62,63,64]
	*XPC/XPA* mutation							
	SNP at *POLG2*	1. POLG2 ↑ 2. Enzymes responsible for mtDNA synthesis and transcription ↑	/	mtDNA copy ↑	OLK	Hyperplasia, mild dysplasia, moderate dysplasia, severe dysplasia, and carcinoma in situ [65].	Erythrosine (photosensitizer): mitochondrial accumulation [66]	[67]
	*COX4I2* and *COX8C* high expression	/	OXPHOS protein markers (like ATP5B and HSP60) ↑	Respiration discovery	BE	Normal squamous epithelium, metaplasia, dysplasia, and esophageal adenocarcinoma	/	[68]
	*BAK1*, *FIS1*, and *SFN* overexpression	/	/	Apoptosis ↓				[69]
mtDNA	Genome instability in mtDNA	/	/	Oxidative stress ↑				[70]
		/	/	/	Gastric precancerous lesion	Chronic gastritis, atrophy, intestinal metaplasia, and dysplasia [71]	/	[72]
		/	/	/	UC	Low-grade dysplasia, high-grade dysplasia [73]	/	[74]
		/	/	/	Cervical dysplasia	/	/	[75]
	*COX* mutation	/	COX ↓	/	DCIS	/	/	[76].
	Mutation in C-tract	/	/	/	OPMD; Head and neck precancerous lesion	/	/	[77,78]
	Mutation in D-loop	/	/	mtDNA copy ↑	OLK	Hyperplasia, mild dysplasia, moderate dysplasia, severe dysplasia, and carcinoma in situ [65].	/	[79]
Non-genetic research	/	/	/	Oxidative stress ↓	OLK, OPL, OSMF	/	/	[80]
			/	Oxidative stress ↑	OSMF	/	/	[81]
			/	Apoptosis ↓	Gastric precancerous lesion	Chronic gastritis, atrophy, intestinal metaplasia, and dysplasia [71]	/	[82]
			/	Warburg effect ↑				[83]
			/	Apoptosis ↑				[84]
			/	Apoptosis ↑	MDS	/	Bortezomib: NF-κB ↓ and mitochondrial related cell death [85]	[86]
			/	Warburg effect ↑	Cervical precancerous lesion	/	ZER: BAX ↑ and BCL-2 ↓ [87]	[88]
			HIF-1α, GLUT1, PKM2, and LDHA, Drp1, OPA1, PGC-1α, UCP2 and mtND1 ↑	1. Warburg effect ↑ 2. Mitochondria number ↑ 3. mtDNA copy ↑	Premalignant colorectal lesion	/	NSAIDs (Diclofenac and Celecoxib): BCL-2 ↓ [89]	[90]
			/	Warburg effect ↑				[91]
			/	Oxidative stress ↑				[92]
			/	Apoptosis ↓				[93]
			/	Mitochondria number ↓				[94]
			/	Mitochondria number ↑				[95]
			/		Liver preneoplastic lesion	/	1. IFN-α2b: BAX ↑ [96]; 2. Combination of celecoxib and synthetic retinoid N-(4-hydroxyphenyl) retinamide (4HPR): BCL-2 ↓ [97]; 3. Glycerol: BAX/BCL-2 ratio ↑, Bad ↑, and PUMA ↑ [98].	[99,100]
			Mitochondrial chaperons ↑	/				[101]
			COX, SDH and glycerol-3-phosphate dehydrogenase ↑	/				[102]
			Change in phospholipid composition of mitochondria	/				[103]
			/	Apoptosis ↑				[104]
				Changes in mitochondrial morphology				[105]
					XP	/	/	[106,107]
					Engineered precancerous epithelial tissue	/	/	[108]
				Warburg effect (↑ or ↓)				[109]
				1. mtDNA copy ↑ 2. Apoptosis ↓	BE	Normal squamous epithelium, metaplasia, dysplasia, and esophageal adenocarcinoma	/	[110]
				Warburg effect (↑ or ↓)				[111]
				Mitochondria number ↑				[112]
				Apoptosis ↓				[113]
				Warburg effect ↑	Pre-malignant prostate lesion	/	/	[114]
				/	Breast precancerous lesion	/	Kaempferol: Drp1 ↑	[115]

Ref, reference; mt, mitochondrial; /, not reported; ↑ increase or promote; ↓ decrease or inhibit; UCP2, uncoupling protein 2; mtND1, mitochondrial dehydrogenase 1; LDHA, lactate dehydrogenase A; TFN, teriflunomide; MDS, myelodysplastic syndromes; PanIN, pancreatic intraepithelial neoplasia; IFN-α2b, interferon alpha 2b; NF-κB, nuclear factor kappa-B; ZER, zerumbone; PUMA, p53 upregulated modulator of apoptosis.

## Supplementary Materials

The following supporting information can be downloaded at: https://www.mdpi.com/article/10.3390/ijms26178537/s1, References [6,7,8,9,10,12,13,15,16,17,20,21,22,34,37,41,43,44,45,48,49,50,51,52,119,120,121,122,123,124,125,126,127,128,129,130,131,132] are cited in the supplementary materials.

## Abbreviations

nDNA	nuclear DNA
ER	endoplasmic reticulum
FAO	fatty acid oxidation
IMM	inner mitochondrial membrane
MAM	mitochondria-associated ER membrane
MMP	mitochondrial membrane potential
MOMP	mitochondrial outer membrane permeabilization
mtQC	mitochondrial quality control
OLK	oral leukoplakia
OMM	outer mitochondrial membrane
OPMD	oral potential malignant disorder
OSCC	oral squamous cell carcinoma
VDAC	voltage-dependent anion channel
