# Supplementary material for: Mitochondria-Associated Pathways in Cancer and Precancerous Conditions: Mechanistic Insights
Source: Int J Mol Sci. 2025 Sep 2;26(17):8537. doi: 10.3390/ijms26178537 (PMC12429017; doi:10.3390/ijms26178537)
Supplement: Supplementary file 1 [file ijms-26-08537-s001.zip › ijms-3810064-supplementary.pdf]

Supporting information of "Mitochondria-Associated Pathways in Cancer and Precancerous Conditions: Mechanistic Insights"

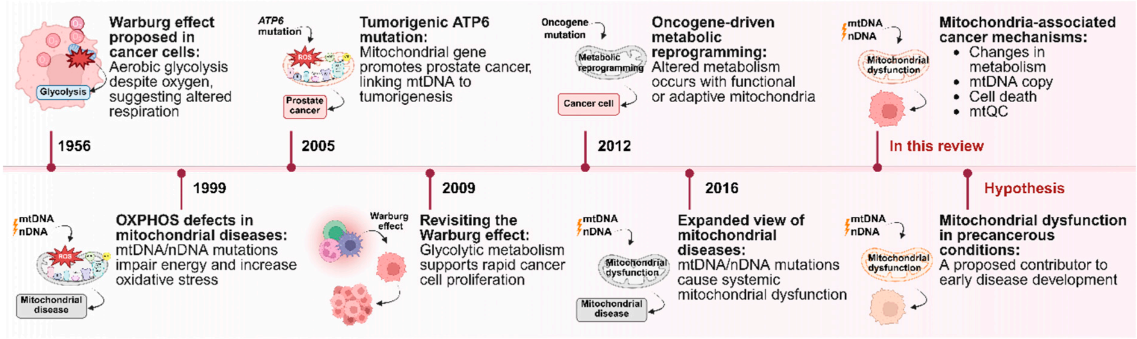

Figure S1. A timeline of key discoveries revealing mitochondria-associated mechanisms in cancer and precancerous conditions.

This figure outlines major milestones in the understanding of mitochondria-related processes in cancer biology. Starting from the Warburg effect in 1956, the timeline highlights advances in recognizing OXPHOS defects in mitochondrial diseases (1999), tumorigenic mtDNA mutations (2005), and evolving views on cancer metabolism (2009–2012). By 2016, mitochondrial dysfunction in cancer was extended beyond metabolism to include mtDNA alterations, quality control, and cell death regulation. This review further integrates evidence suggesting that similar mitochondrial changes may also occur in precancerous conditions, offering new perspectives for diagnosis, therapy, and prevention. mtDNA, mitochondrial DNA; nDNA, nuclear DNA; mtQC, mitochondrial quality control.

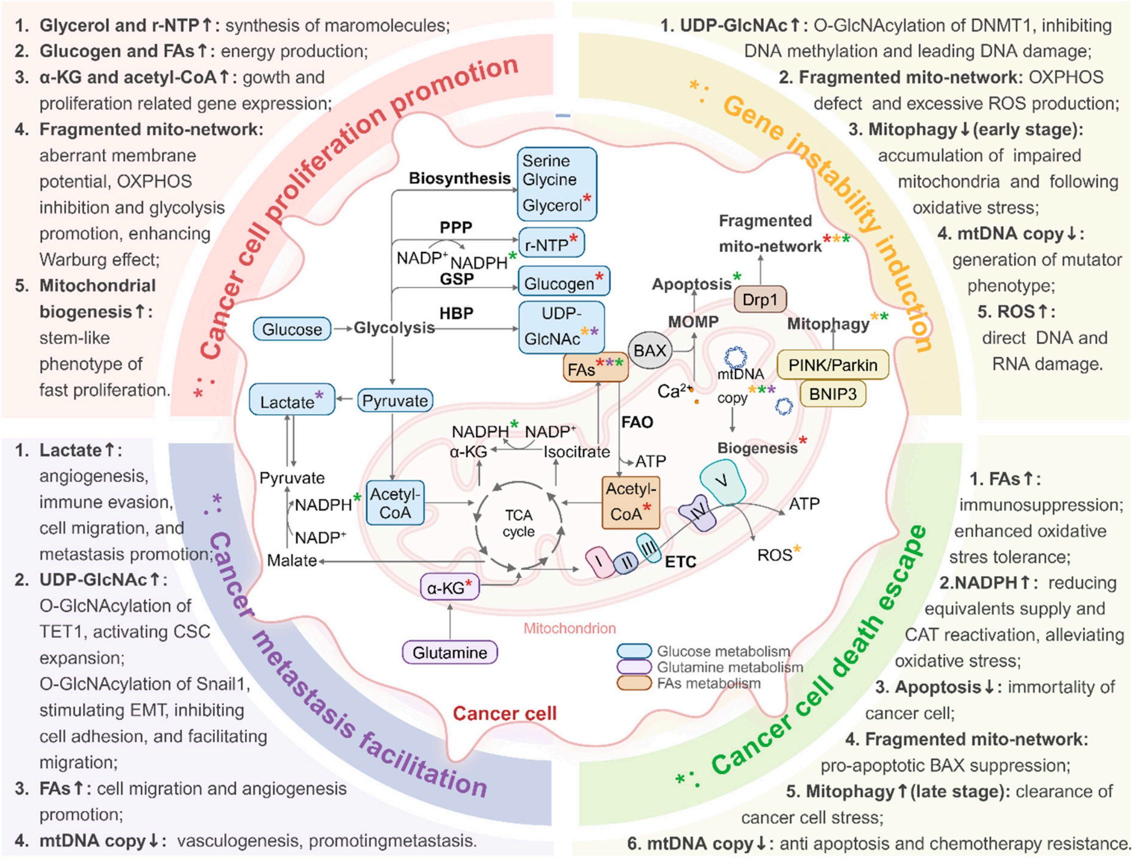

Figure S2. The role of mitochondrial dysfunction in cancer progression: promoting cancer cell proliferation, inducing gene instability, facilitating metastasis, and enabling cancer cell death escape. DNMT1, DNA methyltransferase 1; TET1, tet-methylcytosine dioxygenase 1; CSC, cancer stem cell; EMT, epithelial–mesenchymal transition; CAT, catalase.

Increased glycolysis supplies biomass precursors such as glycerol and r-NTPs for macromolecule synthesis, while glycogen storage supports energy demands, collectively fueling cancer cell proliferation. The glycolytic byproduct UDP-GlcNAc contributes to genetic instability and metastatic potential, while lactate plays a pivotal role in metastasis. Additionally, FAs accumulation supports cancer growth, metastasis, and resistance to apoptosis. The TCA cycle provides acetyl-CoA, *α*-KG, and FAs, which further sustain tumorigenesis. Elevated ROS from the ETC induce DNA damage, exacerbating genetic instability. Meanwhile, NADPH, generated through these pathways, is essential for counteracting oxidative stress, promoting cancer cell survival. mtDNA copy number reduction is linked to the mutator phenotype, metastasis, and apoptosis evasion. Alterations in mitochondrial dynamics, including increased mitochondrial biogenesis and fission, contribute to fragmented mitochondrial networks. Furthermore, **early-stage mitophagy downregulation** promotes genetic instability, whereas **late-stage mitophagy upregulation** facilitates cancer cell survival by mitigating oxidative stress.

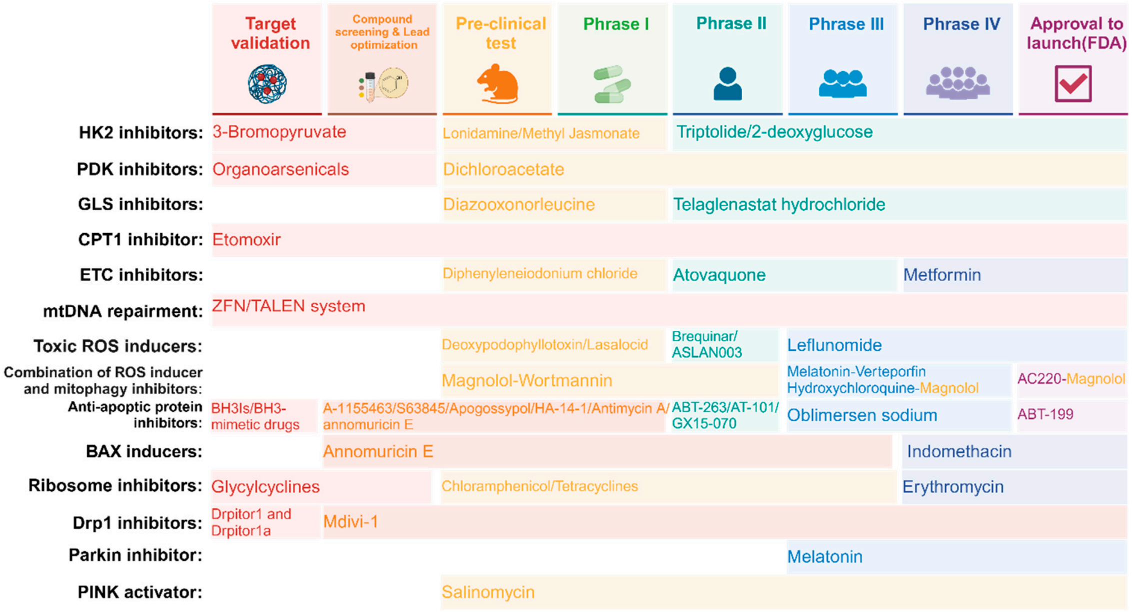

Figure S3. Research stages of mitochondria-targeted drugs for cancer treatment.

The majority of mitochondria-targeted cancer drugs are currently in the validation and preclinical stages of development and clinical use.

Table S1. Gene events and mitochondrial alterations in cancer progression.

	Gene mutation	Pathway	mt-related protein	Cancer type	Result	Alteration of mt-function	Pro-cancer effect	Ref
nDNA	*Pten*	AKT-mTORC1-4EBP1 axis ↑	Translation of HK2 mRNA ↑	Prostate cancer	1. Utilization of glucose ↑<br>2. Mitochondrial membrane stability ↑	1. Warburg effect ↑<br>2. Apoptosis ↓	1. Cancer cell proliferation ↑<br>2. Cancer cell death ↓	[7-9,119-121]
	TP53	miR143 biogenesis ↓	HK2 mRNA stability ↑	Prostate cancer				
		PDK2 ↑	PDH ↓	Breast carcinoma	Pyruvate oxidation ↓	Warburg effect ↑	Cancer cell proliferation ↑	[13]
		SCO2 ↓	Complex IV ↓	Colon cancer	OXPHOS ↓	1. Warburg effect ↑<br>2. Oxidative stress ↑	1. Cancer cell proliferation ↑<br>2. Gene instability ↑	[22]
		c-Myc ↑	CPT1 ↑	/	FAs transport into mitochondria ↑	FAs utilization ↑	Cancer cell proliferation ↑	[122]
		Cooperative action between p53 and p21 ↓	1. BCL-w and BCL-XL ↑<br>2. BAX ↓	Lung and colon cancer	Mitochondrial membrane stability ↑	Apoptosis ↓	Cancer cell death ↓	[34,123,124]
		/	PINK1 ↑	Lung cancer	Protective mitophagy ↑	Oxidative stress ↓ (Late stage)	Cancer cell death ↓	[43,125]
	Park2	/	Parkin ↓	Lung cancer	Protective mitophagy ↓	Oxidative stress ↑ (Early stage)	Gene instability ↑	[45]
	KRAS	AMPK pathway ↑	Drp1 serine 616 phosphorylation ↑	Pancreatic cancer, malignant melanom, ductal carcinoma of the breast and colorectal adenocarcinoma	Mitochondrial fission and fragmented mitochondrial network ↑	1. Oxidative stress ↑<br>2. Warburg effect ↑<br>3. Apoptosis ↓	1. Gene instability ↑<br>2. Cancer cell proliferation ↑<br>3. Cancer cell death ↓	[41,126]
		MAPK pathway ↑	BNIP3 and NIX ↑	Pancreatic ductal adenocarcinoma	Protective mitophagy ↑	Oxidative stress ↓ (Late stage)	Cancer cell death ↓	[44]
	COUP-TFII	COUP-TFII ↑	MPC ↓	Prostate cancer and renal cell carcinoma	Glycolysis ↑	Warburg effect ↑	Cancer cell proliferation ↑	[12,127]
	c-myc (mice)	c-Myc ↑	GLUT1 ↑	/	glucose uptake and metabolism ↑			[6]
	MYC	/	Mitochondrial transcription and mtDNA replication ↑	/	Mitochondrial biogenesis ↑	Mitochondrial number ↑		[37]
		HnRNPI, hnRNPA1, and hnRNPA2 ↑	PKM2/PKM1 ratio ↑	Gliomas	Glycolysis ↑	Warburg effect ↑		[10]
		/	ASCT2, SN2 ↑	Glioblastoma	Glutamine import into mitochondria ↑	Glutamine utilization ↑		[16]
		/	GLS ↑	Prostate cancer	Glutamate conversion into α-KG ↑			[15,128]
	PIK3CA	PDK1-RSK2-ATF4 signalling axis ↑	GPT2 ↑	Colorectal cancer				[17]

	SDHB	/	Complex II ↓	Renal cell carcinoma	OXPHOS ↓	1. Warburg effect ↑ 2. Oxidative stress ↑	1. Cancer cell proliferation ↑ 2. Gene instability ↑	[21]
	GRIM-19	/	Complex I ↓	Renal cell carcinoma				[20]
mtDNA	*ND1, ND2, ND4L, and ND6*	/	ND1, ND2, ND4L, and ND6 (Complex I) ↓	Prostate cancer, acute lymphoblastic leukemia				[48,129]
	COX3	/	COX3 (Complex IV) ↓	/				[48,52,129]
	ATP8	/	ATP6 and ATP8 (Complex V) ↓	Prostate cancer				
	ATP6	/						
		/	PTP ↓	Prostate cancer, acute lymphoblastic leukemia	Mitochondrial Ca^{2+} ↓	Apoptosis ↓	Cancer cell death ↓	
	D-loop region	/	/	Osteosarcoma, Ewing's sarcoma, breast cancer, hepatocellular carcinoma, prostate cancer	/	mtDNA copy ↓	1. Cancer cell death ↓ 2. Gene instability ↑ 3. Cancer metastasis ↑	[49-51,130-132]

Ref, reference; mt, mitochondrial; /: Not reported; ↑, increase or promote; ↓, decrease or inhibit.
